# Efficient multimode Wigner tomography

**DOI:** 10.1038/s41467-024-48573-x

**Published:** 2024-05-16

**Authors:** Kevin He, Ming Yuan, Yat Wong, Srivatsan Chakram, Alireza Seif, Liang Jiang, David I. Schuster

**Affiliations:** 1 James Franck Institute, University of Chicago, Chicago, IL 60637 USA; 2 Department of Physics, University of Chicago, Chicago, IL 60637 USA; 3 Pritzker School of Molecular Engineering, University of Chicago, Chicago, IL 60637 USA; 4 Department of Physics and Astronomy, Rutgers University, Piscataway, NJ 08854 USA

**Keywords:** Quantum information, Quantum simulation

## Abstract

Advancements in quantum system lifetimes and control have enabled the creation of increasingly complex quantum states, such as those on multiple bosonic cavity modes. When characterizing these states, traditional tomography scales exponentially with the number of modes in both computational and experimental measurement requirement, which becomes prohibitive as the system size increases. Here, we implement a state reconstruction method whose sampling requirement instead scales polynomially with system size, and thus mode number, for states that can be represented within such a polynomial subspace. We demonstrate this improved scaling with Wigner tomography of multimode entangled W states of up to 4 modes on a 3D circuit quantum electrodynamics (cQED) system. This approach performs similarly in efficiency to existing matrix inversion methods for 2 modes, and demonstrates a noticeable improvement for 3 and 4 modes, with even greater theoretical gains at higher mode numbers.

## Abstract (web summary)

Standard ways of characterising quantum states incur exponential overhead. Here, the authors consider the task of reconstructing density matrices of multimode continuous variable systems, and demonstrate a method which scales polynomially with the system size, provided the state lies in a polynomial dimensional subspace.

## Introduction

Quantum state tomography (QST) is the process of determining the quantum state of a system, and is a crucial part of certifying operations and characterizing processes in quantum information science. In its conventional formulation, obtaining full state information has a processing and measurement requirement that scales exponentially with the size of the system^1,2^. However, physical states of interest typically have some structure that we can exploit to simplify the measurement complexity. Direct fidelity estimation (DFE) is a technique that utilizes this, and has been applied to matrix product states or stabilizer states in many-qubit systems^2–5^ to efficiently produce partial information about the system state. In the remainder of this work, we refer to such states as DFE-efficient.

Efficient QST is especially relevant in continuous variable systems with bosonic cavity modes, whose Hilbert spaces are arbitrarily large. These systems have applications in error correction codes^6–8^, quantum optics^9^, quantum simulation^10^, and quantum information processing^11^. For a single mode, full state information is obtained by measuring operators like the Wigner operator^12^ or Q function operator at different mode displacements^13^. Efficient QST in the multimode case is much more challenging. Even with techniques like compressed sensing^14–16^, the sampling and number of measurements required can still scale exponentially with the number of modes. Several efforts use multiple cavities to propose or produce increasingly complex states like multimode cat states^17^, W states^18^, multimode GKP states^19^, GHZ states^20^, and other multimode Fock state superpositions^21,22^ that have a variety of applications in quantum error correction and logical encodings, as well as quantum simulation. In particular, W states have unique multipartite entanglement—demonstrated with entanglement witnesses^23,24^—and protection against photon loss that gives them applications in quantum communication. Some proposed theoretical methods are able to extract multimode state information while circumventing the exponential scaling of observation number with the number of modes. These include techniques that apply additional unitaries between modes as part of the measurement process, perform targeted measurements with polynomial post-processing^25^, make use of ancillary modes and a known excitation number^26^, or apply operators based on excitation counting^13^. In our case, to obtain full state information, we instead use a technique that in principle only requires local operations to directly measure and reconstruct the density matrices of potentially mixed states confined in a subspace of interest.

In this work, we use the Direct Extraction of (Density) Matrix Elements from Subspace Sampling Tomography (DEMESST) method to reconstruct quantum state density matrices, and compare its performance with an optimized QST method^18,27^. DEMESST applies when an unknown state lies in a polynomial dimensional subspace. When this subspace is spanned by a set of states obtained by acting finite local operations on a DFE-efficient state, the operations required to implement DEMESST are local as well^28^. Under these conditions, DEMESST has a polynomially scaling sampling requirement. For certain multimode cavity states, the total measurement number will therefore depend polynomially on the number of modes, rather than exponentially. With DEMESST, we individually sample measurements for each basis operator in a polynomial subspace, and subsequently reconstruct a density matrix by combining them. Additionally, we implement this method with Wigner operators and Wigner tomography, thus performing the measurements with local operations on the modes and eliminating errors that may be associated with multimode unitaries or beamsplitters. With this approach, and without making prior assumptions about the populations or phases of the state components, we measure the density matrices of W states prepared in up to 4 bosonic modes on a superconducting cQED system, which is beyond existing demonstrations and advances the state of the art.

## Results

### The DEMESST method

**Fig. 1:**
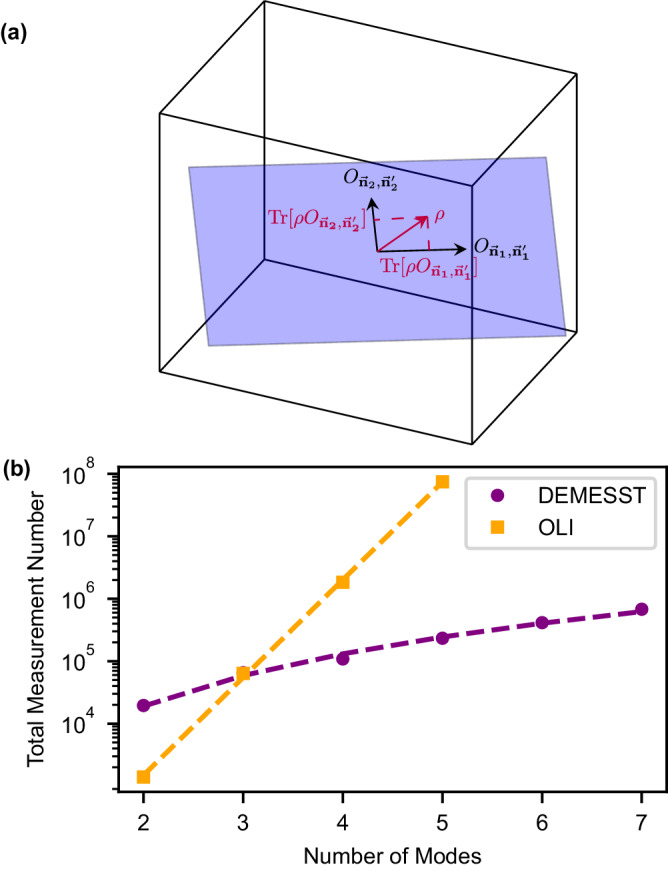
Theoretical comparison and schematic representation of tomography methods. **a** Schematic representing the DEMESST method. Rather than sampling an entire multimode operator space (3D space), if a state lives in some number of polynomial subspaces (blue 2D plane), we restrict the sampling to each of those instead. The {*O*} basis operators are of the form ${O}_{\overrightarrow{{{{{{{{\bf{n}}}}}}}}},{\overrightarrow{{{{{{{{\bf{n}}}}}}}}}}^{{\prime} }}=\left\vert \overrightarrow{{{{{{{{\bf{n}}}}}}}}}\right\rangle \left\langle {\overrightarrow{{{{{{{{\bf{n}}}}}}}}}}^{{\prime} }\right\vert$ for generic basis states $\left\vert \overrightarrow{{{{{{{{\bf{n}}}}}}}}}\right\rangle,\left\vert {\overrightarrow{{{{{{{{\bf{n}}}}}}}}}}^{{\prime} }\right\rangle$ (see Supplementary Note 4). Assuming an orthonormal basis, the state *ρ* is given by $\rho={\sum }_{{\overrightarrow{{{{{{{{\bf{n}}}}}}}}}}_{{{{{{{{\bf{1}}}}}}}}},{\overrightarrow{{{{{{{{\bf{n}}}}}}}}}}_{{{{{{{{\bf{2}}}}}}}}}}\,{{\mbox{Tr}}}\,[\rho {O}_{{\overrightarrow{{{{{{{{\bf{n}}}}}}}}}}_{{{{{{{{\bf{2}}}}}}}}},{\overrightarrow{{{{{{{{\bf{n}}}}}}}}}}_{{{{{{{{\bf{1}}}}}}}}}}]{O}_{{\overrightarrow{{{{{{{{\bf{n}}}}}}}}}}_{{{{{{{{\bf{1}}}}}}}}},{\overrightarrow{{{{{{{{\bf{n}}}}}}}}}}_{{{{{{{{\bf{2}}}}}}}}}}$. This improves the overall efficiency of the sampling, especially for states with support across large numbers of modes. In practice, we use Hermitian {*O*} that are accessible through experiment. **b** Number of measurements required for the DEMESST (purple, circles) and OLI (orange, squares) methods to reach a 90% state reconstruction fidelity on W states of up to 7 modes, assuming perfect state preparation. Dashed lines indicate fits to exponential and polynomial functions $y=\exp (a+bx)$ with *a* = 0.1, *b* = 3.6 and $y=\exp (a+b\log (x))$ with *a* = 7.9, *b* = 2.8, respectively. OLI scales exponentially with the number of modes *M*, while DEMESST scales only polynomially.

DEMESST scales polynomially with mode number for states that have support in a polynomial subspace of DFE-efficient basis operators^3^ (for example, states with a known maximum excitation number in the Fock basis). This is accomplished by leveraging that prior information, and rather than sampling all basis operators of the Hilbert space, only sampling those that are expected to support the state. This is especially advantageous when the subspace that an expected state lives in is much smaller than the full space. DEMESST allows us to individually sample density matrix elements in a subspace of interest, and applies to both discrete qubit and continuous cavity systems. This is illustrated schematically in Fig. 1a. We reconstruct a density matrix *ρ* in a polynomial subspace by independently measuring basis operators and the corresponding matrix elements for each projection of *ρ* within the subspace, through methods similar to DFE^3,4^, without introducing statistical bias, thus ensuring convergence to the true values (see Supplementary Notes 3 and 4). The reconstructed state is given by

1 $$\rho=\left[\begin{array}{ccc}\,{{\mbox{Tr}}}\,\left[\rho {O}_{{\overrightarrow{{{{{{{{\bf{n}}}}}}}}}}_{1},{\overrightarrow{{{{{{{{\bf{n}}}}}}}}}}_{{{{{{{{\bf{1}}}}}}}}}}\right]&\,{{\mbox{Tr}}}\,\left[\rho {O}_{{\overrightarrow{{{{{{{{\bf{n}}}}}}}}}}_{2},{\overrightarrow{{{{{{{{\bf{n}}}}}}}}}}_{1}}\right]&\ldots \\ &\,{{\mbox{Tr}}}\,\left[\rho {O}_{{\overrightarrow{{{{{{{{\bf{n}}}}}}}}}}_{2},{\overrightarrow{{{{{{{{\bf{n}}}}}}}}}}_{2}}\right]\\ \vdots &&\ddots \end{array}\right]$$

With this approach, we avoid extracting irrelevant information about states outside the subspace of interest, thereby lowering the number of measurements required for an accurate result. For example, if we know a 3-mode state has a maximum of 2 photons (*M* = 3, *d* = 3 for 0, 1, or 2 photon population), we reconstruct it with DEMESST by measuring the matrix elements associated with that subspace, namely the one formed by $\left\vert 000\right\rangle,\left\vert 001\right\rangle,\left\vert 011\right\rangle,\left\vert 002\right\rangle$, and permutations. We eliminate unnecessary sampling of higher photon number states like $\left\vert 111\right\rangle$ or $\left\vert 012\right\rangle$ and beyond. Additionally, we build upon existing work^2,3^ by developing and applying this approach to QST of bosonic modes and arbitrary mixed states rather than qubits and pure states. Here, we implement DEMESST on a multimode cavity system with Wigner tomography.

### Optimized Wigner tomography (OLI)

Wigner tomography uses measurements of the Wigner operator ${{{{{{{\mathcal{W}}}}}}}}(\overrightarrow{{{{{{{{\boldsymbol{\alpha }}}}}}}}})={{{{{{{\mathcal{D}}}}}}}}(\overrightarrow{{{{{{{{\boldsymbol{\alpha }}}}}}}}})\Pi {{{{{{{\mathcal{D}}}}}}}}(-\overrightarrow{{{{{{{{\boldsymbol{\alpha }}}}}}}}})$ acting on a bosonic state *ρ* to reconstruct it. Here, ${{{{{{{\mathcal{D}}}}}}}}(\overrightarrow{{{{{{{{\boldsymbol{\alpha }}}}}}}}}){=\bigotimes }_{i}{{{{{{{\mathcal{D}}}}}}}}({\alpha }_{i})$ is the displacement operator and Π is a parity measurement. Existing inversion-based Wigner tomography methods operate by taking the Wigner functions of a set of displacements $\{\overrightarrow{{{{{{{{\boldsymbol{\alpha }}}}}}}}}\}$ to construct a measurement matrix ${{{{{{{\mathcal{M}}}}}}}}$ that maps to states as $\overrightarrow{{{{{{{{\bf{x}}}}}}}}}={{{{{{{\mathcal{M}}}}}}}}\left.\left| \rho \right\rangle \right\rangle$, where $\left.\left| \rho \right\rangle \right\rangle$ is the vectorized form of *ρ*. Inverting ${{{{{{{\mathcal{M}}}}}}}}$ to ${{{{{{{{\rm{minimize}}}}}}}}}_{\rho }| | {{{{{{{\mathcal{M}}}}}}}}\left.\left| \rho \right\rangle \right\rangle -\overrightarrow{{{{{{{{\bf{x}}}}}}}}}| |$ allows us to determine the physical (unit trace and positive semidefinite) *ρ* that was most likely to have produced $\overrightarrow{{{{{{{{\bf{x}}}}}}}}}$. The set $\{\overrightarrow{{{{{{{{\boldsymbol{\alpha }}}}}}}}}\}$ is optimized by minimizing the condition number (the ratio of largest to smallest eigenvalue) of ${{{{{{{\mathcal{M}}}}}}}}$ and thus the error magnification, using the techniques presented in refs. ^18,27^. We refer to this method as Optimized Linear Inversion (OLI). In this approach, to make the problem tractable, we choose a cutoff dimension *d* to truncate the Hilbert space. Reconstructing *ρ* for a single mode therefore requires at least *d*^2^ measurements to determine each density matrix parameter. For multiple modes, the size of the Hilbert space and thus the number of required measurements will scale exponentially, requiring at least *d*^2*M*^ observations for *M* modes. We compare OLI with DEMESST by testing their performance on experimentally prepared W states.

### Testing tomography sampling with W states

W states are excellent candidates for testing our Wigner tomography sampling methods. For 2 modes, an ideal W state is given by $\left\vert {{{\mbox{W}}}}_{2}\right\rangle=(\left\vert 10\right\rangle+{e}^{i\phi }\left\vert 01\right\rangle )/\sqrt{2}$. For 3 modes, $\left\vert {{{\mbox{W}}}}_{3}\right\rangle=(\left\vert 100\right\rangle+{e}^{i{\phi }_{1}}\left\vert 010\right\rangle+{e}^{i{\phi }_{2}}\left\vert 001\right\rangle )/\sqrt{3}$, and similarly for 4 modes, $\left\vert {{{\mbox{W}}}}_{4}\right\rangle=(\left\vert 1000\right\rangle+{e}^{i{\phi }_{1}}\left\vert 0100\right\rangle+{e}^{i{\phi }_{2}}\left\vert 0010\right\rangle+{e}^{i{\phi }_{3}}\left\vert 0001\right\rangle )/\sqrt{4}$. Here, the *ϕ*_*j*_’s are a priori unknown phases on each of the state components, and are determined through measurement. Additionally, due to imperfect state preparation, we make no assumptions about the component populations and precisely measure each one separately. We further generalize this: rather than restricting ourselves to the pure states ${{{\mbox{W}}}}_{j}=\left\vert {{{\mbox{W}}}}_{j}\right\rangle \left\langle {{{\mbox{W}}}}_{j}\right\vert$, we measure the full, possibly mixed density matrices. W states are suitable representative states because they are irreducible multimode states that generalize straightforwardly to arbitrary numbers of modes, and have a well-defined photon number. We prepare them easily using photon blockade^18,29–31^.

### Simulated performance of OLI and DEMESST

We first investigate the simulated theoretical performance of DEMESST and OLI on *M*-mode W states. Assuming perfect state preparation, we compare the number of observations required to accurately reconstruct the W state with 90% fidelity. This is shown in Fig. 1b. Inversion-based methods like OLI have a sampling requirement that scales exponentially with mode number *M*. In contrast, the DEMESST method scales polynomially with the subspace dimension and thus *M* when we have a fixed maximum photon number, demonstrating an advantage that increases with *M*. For two modes, OLI performs better due to measurement sampling overhead associated with the DEMESST approach (see Supplementary Notes 4 and 7). However, for larger *M*, DEMESST requires fewer measurements to converge to the same level of fidelity, and scales much more efficiently than OLI. We proceed to demonstrating this expected behavior in experiment.

### Pulse sequences and cQED hardware

**Fig. 2:**
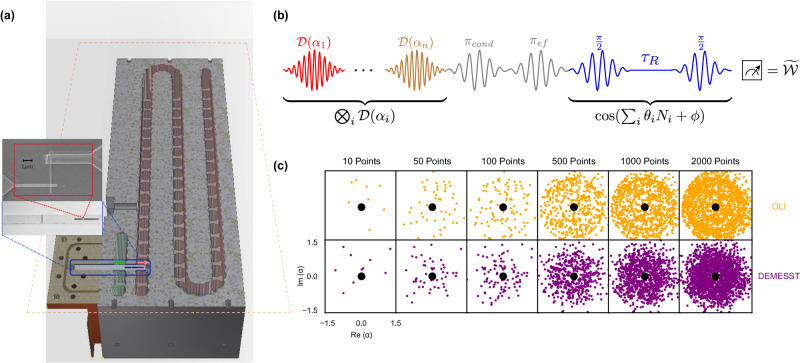
Experimental system and scheme. **a** Adapted with permission from ref. ^32^. Copyrighted by the American Physical Society. **a** Schematic of the multimode 3D cQED system with rectangular flute-style storage and readout cavities that both couple to a transmon circuit. Experimental drives are input through a drive pin on the readout cavity, or through a drive pin directly on the storage cavity. **b** Wigner tomography pulse sequence. Initial cavity displacements and a final generalized multimode parity measurement implement the tomography, while optional conditional *π* pulses are used to target specific modes and take the transmon portion of their joint transmon-cavity states to $\left\vert f\right\rangle$ and out of the qubit subspace in the DEMESST approach (see Supplementary Note 5). An additional angle *ϕ* is applied between the *π*/2 pulses of the parity measurement to rotate the generalized Wigner function onto the real axis. **c** Cavity displacement plots for the OLI (orange, above) and DEMESST (purple, below) sampling methods. The OLI has ring features corresponding to measurement of all Fock states up to a cutoff, while DEMESST has points more densely located in the phase space based on the basis state being measured.

We generate W states and implement DEMESST and OLI on a superconducting 3D cQED platform. The system consists of a transmon qubit coupled to a 3D readout cavity and a 3D multimode storage cavity, like the one presented in ref. ^32^. A schematic of this hardware setup is shown in Fig. 2a. The single storage cavity supports many bosonic cavity modes at roughly equally spaced microwave frequencies. The transmon allows for universal control of the cavity modes and also mediates interactions like photon blockade^29,31^ between the storage modes. We use four of the modes to prepare our multimode W states. We also use the transmon to implement the parity measurements necessary for the multimode Wigner tomography.

The tomography sampling methods use the same generalized Wigner tomography sequence, where each cavity mode is displaced before performing a generalized parity measurement on the transmon^18^. This procedure allows us to perform multimode tomography measurements despite having unequal dispersive shifts *χ*_*m*_ for different modes, without requiring *χ* engineering techniques^33,34^ or additional control pulses. This pulse sequence is shown in Fig. 2b. For the DEMESST method, we may also include a *π*_*ge*_ pulse conditioned on certain cavity populations followed by an *π*_*ef*_ pulse on the transmon. These pulses transfer the transmon portion of the joint transmon-cavity state of one or more of the cavity modes to the $\left\vert f\right\rangle$ (second excited) level and outside the $\left\vert g\right\rangle -\left\vert e\right\rangle$ qubit space used for the Wigner tomography. This effectively removes those modes from the measurement and reduces the sampling requirement. We apply this technique when the basis operator being measured has one or more cavity modes in vacuum. For example, to sample the 3-mode matrix element $\left\vert 001\right\rangle \left\langle 010\right\vert$, we take the transmon state of the first mode from $\left\vert g\right\rangle$ to $\left\vert f\right\rangle$ and reduce the sampling to the 2-mode $\left\vert 01\right\rangle \left\langle 10\right\vert$ operator. This operation allows us to reduce the size of the sampling problem for that element to that of a lower number of modes (see Supplementary Note 5).

### OLI and DEMESST fidelities and matrix distances

We first compare the state reconstructed in experiment using the OLI method to simulation, which we use as a baseline for later comparison to DEMESST. With OLI, we find experimentally prepared W state fidelities that are in good agreement with the simulated fidelities, as shown in Table 1. The simulations include decoherence and state preparation errors, such as leakage outside of the blockaded subspace. We now continue to the DEMESST performance.

**Table 1:** OLI and DEMESST tomography results

	Simulated Fidelity	OLI Fidelity	DEMESST Fidelity	OLI:DEMESST Distance
2-mode	0.971	0.966(5)	0.96(1)	0.96(2)
3-mode	0.956	0.949(4)	0.955(4)	1.55(10)
4-mode	0.912	0.912(7)	0.911(7)	2.3(2)

Simulated and measured Wigner tomography fidelities for *M*-mode W states of varying size. The simulated fidelities are obtained by comparing ideal W states to the states obtained from Lindblad master equation simulations with the experimental control drives. The fidelities are in good agreement, and the Frobenius norm matrix distance ratio is compatible with exponential improvement for DEMESST vs. OLI as *M* increases.

For the DEMESST approach, we reconstruct the 2–4 mode density matrices by measuring Wigner operators corresponding to multimode Fock basis states with up to 2 photons. Even though W states have at most one photon, we measure two-photon operators to capture imperfect state preparation errors. These observations directly provide the values of each density matrix element. From the density matrix, we obtain the component populations and phase angles *ϕ*_*j*_ of our prepared W states by calculating the phase angle value that best matches the resulting data. These angles are then verified to match with the ones obtained from the OLI approach.

**Fig. 3:**
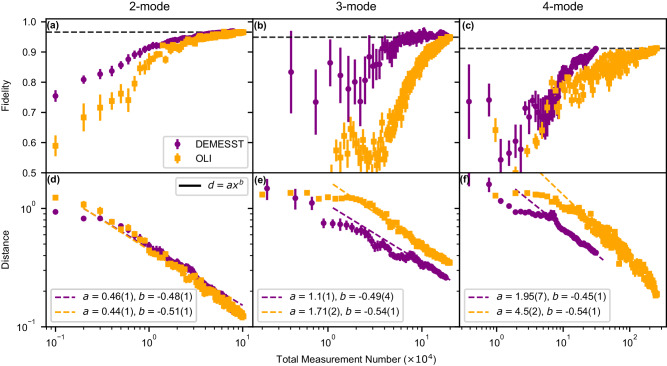
Tomography fidelity and matrix distances for DEMESST and OLI sampling methods. Sampling was performed on approximate entangled W states of 2--4 modes. The top (**a**)–(**c**) show fidelities to an ideal (exactly equal population coefficients) W state for 2–4 modes, with dashed horizontal lines indicating the final converged fidelity obtained from the OLI method. These final fidelities are, for 2–4 modes, 0.966 ± 0.005, 0.949 ± 0.004, and 0.912 ± 0.007 for OLI and 0.96 ± 0.01, 0.954 ± 0.004, and 0.911 ± 0.007 for DEMESST and are in good agreement. The bottom (**d**)–(**f**) show Frobenius norm matrix distances between the state at a given measurement number versus the final measured state. Error bars indicate the standard error. The rates of convergence are close to 1/$\sqrt{x}$ or a power of −0.5, as expected. As the mode number increases, the DEMESST method performs increasingly more efficiently by requiring fewer measurements to reach a given level of convergence or error threshold.

We quantify the performance of the DEMESST and OLI sampling methods versus total measurement number with two metrics: reconstructed state fidelity and Frobenius norm matrix distance. The fidelities are computed with respect to an ideal W state, while the matrix distances are calculated with respect to the experimentally prepared state reconstructed at the maximum measurement number. These results are shown in Fig. 3. The final fidelities obtained from the DEMESST approach are presented in Table 1, and are consistent with the OLI results. For the 2-mode W state, the two methods perform similarly, while for 3 and 4 modes, DEMESST performs better than OLI with faster convergence to the final state.

This improvement is most evident in the matrix distance comparisons. The distances are computed using the Frobenius norm. The behavior of both sampling methods is nearly identical for the 2-mode W state. However, for the 3-mode case, DEMESST has noticeably faster convergence versus total measurement number *x*, as the matrix distance *d* to the final state is smaller, as seen by the fit coefficient to *d* = *ax*^*b*^ (*a* = 1.1 ± 0.1 for DEMESST versus 1.71 ± 0.02 for OLI). This effect is further enhanced in the 4-mode case. The ratio of these fit values scales roughly geometrically, as shown in Table 1, reflecting the fact that OLI scales exponentially while DEMESST only scales polynomially versus total measurement number. In all cases, the distances fall off roughly as *x*^−1/2^, as expected.

### Self-consistency of DEMESST

An advantage of the DEMESST sampling method compared to OLI is its self-consistency. Individual density matrix elements for any multimode state are measured independently, without needing to choose a cutoff maximum photon number or Hilbert space size that could subject the reconstructed state to inversion errors. This eliminates the risk of obtaining an inaccurate tomography result if, for example, the prepared state contains population beyond the space spanned by our chosen basis during OLI sampling.

**Fig. 4:**
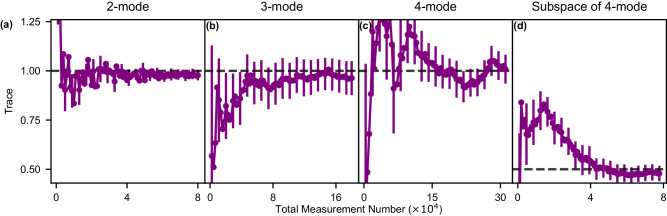
Trace verification for the DEMESST sampling method. Trace versus point number for prepared (**a**) 2-mode, (**b**) 3-mode, and (**c**) 4-mode W states. Error bars are shown for every fourth point, as well as the final one, and indicate the standard error. They are obtained from comparing the traces from individual sets of measurements. As expected, the traces converge to values near unity. **d** Trace versus point number when measuring only a 2-mode subspace of a prepared 4-mode W state. Due to only measuring half of the populated space, the trace converges to 0.5. This demonstrates that the DEMESST sampling method is self-consistent and does not depend on the chosen measurement subspace.

We verify that the DEMESST tomography sampling method leads to self-consistent measurement results. We check the traces of our prepared W states and compare them with unity. This allows us to confirm that our prepared state indeed lives in our chosen, measured Hilbert space. The results are shown in Fig. 4 as average trace versus observation number. Like before, the averages are taken over 10 independent sets of 10 measurement repetitions for each sampled displacement. We find that in all cases ((a)–(c)), the observed traces are near one. We attribute large deviations from unity at low measurement numbers to noise and statistics, and attribute the final traces being slightly less than one to imperfect state preparation that produces population outside the measured subspace. We perform a further check by considering only a 2-mode subspace of a prepared 4-mode W state. This is shown in Fig. 4d. As expected, the measured trace converges to a value near 0.5, as we are effectively only observing half of the total state population. This demonstrates that the DEMESST method provides accurate results for each basis operator independently. In particular, we can identify when we have measured insufficient basis elements to fully characterize a state, such as when the state lives partially (or entirely) outside the corresponding space, which is a useful capability in itself.

## Discussion

In summary, we have applied the DEMESST sampling method to characterize multimode cavity states with Wigner tomography. DEMESST is most appropriate for multimode states that have population contained in a subspace of DFE-efficient elements of an overall Hilbert space, and outperforms traditional optimized inversion-based methods by scaling polynomially rather than exponentially with mode number. We observe this improvement for W states on 3 and 4 modes. Here, we have presented comparisons using the multimode Fock basis on multimode W states, but DEMESST also applies to different bases that more readily support other states; this tomography method can even be used for DFE by choosing as a basis the intended target state. While Wigner tomography was presented in this work, the method also operates beyond the bosonic Wigner function, and works for both continuous and discrete systems. This approach can in principle operate without coupling gates between modes, such as when each mode has its own transmon for performing parity measurements, which would be useful for calibrating entangled states over distributed quantum networks. Ultimately, the DEMESST sampling method enables efficient reconstruction of certain large multiqubit or multimode states, which will be advantageous as the size of quantum hardware increases and more complicated states are generated and applied for quantum simulation, bosonic logical state encoding, and error correction.

## Methods

### Measurement statistics

Error bars shown in Figs. 3 and 4 are obtained from the results of 10 independent sets of 10 repetitions of tomography measurements for each sampled displacement, and indicate the standard error. The number of distinct displacements for the OLI method therefore equals the Total Measurement Number shown on the x-axis in Fig. 3 divided by 10. For the DEMESST method, the number of distinct displacements for each separate basis element is further divided by the number of such basis or density matrix elements.

In the matrix distance plots in Fig. 3, the final state density matrix against which the distances are computed is obtained by considering all 100 measurement repetitions, rather than sets of 10, which is why the final distances do not completely vanish.

### Supplementary information

Supplementary Information

Peer Review File

## Data Availability

The data used in this study is available in the Figshare database at 10.6084/m9.figshare.24158481.

## Code availability

The code used in this study is available in the Figshare database at 10.6084/m9.figshare.24158481.
