# Supplementary material for: Efficient multimode Wigner tomography
Source: Nat Commun. 2024 May 16;15:4138. doi: 10.1038/s41467-024-48573-x (PMC11099137; doi:10.1038/s41467-024-48573-x)
Supplement: Supplementary file 1 — Supplementary Information [file 41467_2024_48573_MOESM1_ESM.pdf]

Supplementary Information: Efficient multimode Wigner tomography

Supplementary Note 1 – Calibration of the multimode Hamiltonian

Like in [1], the Hamiltonian of the multimode cQED system realized by the transmon and the storage modes is:

$$H = \omega_q |e\rangle\langle e| + \sum_{m=0}^{N-1}\{\omega_m a_m^\dagger a_m + \chi_m a_m^\dagger a_m |e\rangle\langle e| + \frac{k_m}{2} a_m^\dagger a_m (a_m^\dagger a_m - 1)\} + \sum_{n\neq m} k_{mn} a_m^\dagger a_m a_n^\dagger a_n, \quad (1)$$

where $\omega_q/(2\pi) = 4.96$ GHz is the frequency of the transmon $|g\rangle$ - $|e\rangle$ transition, $\omega_m/(2\pi)$ ranging from $5.72-6.47$ GHz are the cavity mode frequencies, $\chi_m/(2\pi)$ ranging from -1.64 to -0.91 MHz are the dispersive shifts, k_m the self-Kerr shift of each mode, and k_{mn} the cross-Kerr interactions between the modes. The Kerr nonlinearities range from -6 to $+9$ kHz. Parameter values are determined with the processes described in the supplement of [1]. A summary of the system parameters (updated from [1]) used in the experiment, as well as Liouvillian terms corresponding to transmon and cavity decoherence and decay, is provided in STable 1. The 2-mode experiments used the last two modes in the table, the 3-mode experiments used the last three modes, and the 4-mode experiments used all four of the modes.

Supplementary Note 2 – The Wigner function and its generalization

The Wigner function is the quasiprobability distribution of a state in phase space, and is one of the most important functions in the field of quantum optics. For a single-mode state ρ, the Wigner function is defined as [3]

$$W_\rho(\alpha) = 2\mathrm{Tr}[\rho D(\alpha) e^{i\pi a^\dagger a} D^\dagger(\alpha)], \quad (2)$$

where $D(\alpha) = e^{\alpha a^\dagger - \alpha^* a}$ is the displacement operator. We can see that $W_\rho(\alpha)$ is proportional to the expectation value of the parity operator with the state ρ displaced by complex amplitude $-\alpha$. Similarly, we can introduce the Wigner function for an operator O with finite Frobenius norm (F-norm) ($||O||_F = \sqrt{\mathrm{Tr}[O^\dagger O]} < +\infty$) by substituting the state ρ with the operator O in Supplementary Eqn. (2).

The Wigner function of a multimode M-mode state ρ can be expressed as

$$W_\rho(\vec{\alpha}) = 2^M \mathrm{Tr}[\rho D(\vec{\alpha}) e^{i\pi\sum_{m=1}^M a_m^\dagger a_m} D^\dagger(\vec{\alpha})], \quad (3)$$

where $D(\vec{\alpha}) = \prod_{m=1}^M e^{\alpha_m a_m^\dagger - \alpha_m^* a_m}$. As described in the main text, due to the different dispersive shifts of our cavity modes, we instead implement a generalized parity operator [1]. We can express the corresponding generalized version of the Wigner function, as introduced in [4], as

$$W_\rho(\vec{\alpha}, \vec{\theta}) = \frac{2^M \mathrm{Tr}[\rho D(\vec{\alpha}) e^{i\sum_{m=1}^M \theta_m a_m^\dagger a_m} D^\dagger(\vec{\alpha})]}{\prod_{m=1}^M [1 + i\cot(\theta_m/2)]}, \quad (4)$$

where θ_m can be different and need not equal π for the M modes. We also denote

$$\tilde{W}_\rho(\vec{\alpha}, \vec{\theta}) = \mathrm{Tr}[\rho D(\vec{\alpha}) e^{i\sum_{m=1}^M \theta_m a_m^\dagger a_m} D^\dagger(\vec{\alpha})], \quad (5)$$

where $\tilde{W}_\rho(\vec{\alpha}, \vec{\theta})$ can in general be a complex number, since $e^{i\sum_{m=1}^M \theta_m a_m^\dagger a_m}$ is no longer Hermitian. Also, $|\tilde{W}_\rho(\vec{\alpha}, \vec{\theta})| \leq 1$. We can see from the form of Supplementary Eqn. (5) that $\tilde{W}_\rho(\vec{\alpha}, \vec{\theta}) = \tilde{W}^*_{\rho^\dagger}(\vec{\alpha}, -\vec{\theta})$. This generalized Wigner function $\tilde{W}_\rho(\vec{\alpha}, \vec{\theta})$ is what we experimentally measure. In the next note, we show that $\tilde{W}_\rho(\vec{\alpha}, \vec{\theta})$ for quantum states plays a similar role as the usual Wigner function (Supplementary Eqn. (3)) when calculating the expectation values with other operators.

Supplementary Note 3 – Estimating expectation values with Wigner sampling

In this note, we discuss the sampling method for estimating the expectation value of the Wigner function with an operator with finite F-norm O. We then analyze the overhead required to reach a certain accuracy threshold.

We start with the identity that reflects the relationship between expectation values and the Wigner function:

$$\mathrm{Tr}[\rho O] = \int \frac{\mathrm{d}^{2M}\vec{\alpha}}{\pi^M} W_\rho(\vec{\alpha}) W_O(\vec{\alpha}). \quad (6)$$

For the generalized Wigner function, we have a similar expression:

$$\begin{aligned}\mathrm{Tr}[\rho O] &= \int \frac{\mathrm{d}^{2M}\vec{\alpha}}{\pi^M} W_\rho(\vec{\alpha}, -\vec{\theta}) W_O(\vec{\alpha}, \vec{\theta}) \\ &= C_M \int \mathrm{d}^{2M}\vec{\alpha}\, \tilde{W}_\rho(\vec{\alpha}, -\vec{\theta}) \tilde{W}_O(\vec{\alpha}, \vec{\theta}),\end{aligned} \quad (7)$$

where $C_M = \prod_{m=1}^M [2(1-\cos\theta_m)/\pi]$.

The equations above have been applied for direct fidelity estimation between an experimentally prepared state ρ and a target pure state σ [5]. For example, we can rewrite Supplementary Eqn. (7) as

$$\begin{aligned}F(\rho, \sigma) = \mathrm{Tr}[\rho\sigma] &= C_M \int \mathrm{d}^{2M}\vec{\alpha}\, |\tilde{W}_\sigma(\vec{\alpha}, \vec{\theta})|^2 \frac{\tilde{W}_\rho(\vec{\alpha}, -\vec{\theta})}{\tilde{W}_\sigma(\vec{\alpha}, -\vec{\theta})} \\ &= \int \mathrm{d}^{2M}\vec{\alpha}\, p_{W^2}(\vec{\alpha}) \frac{\mathrm{Re}[e^{i\phi(\vec{\alpha})} \tilde{W}_\rho(\vec{\alpha}, -\vec{\theta})]}{|\tilde{W}_\sigma(\vec{\alpha}, -\vec{\theta})|},\end{aligned} \quad (8)$$

Parameter	Hamiltonian/Liouvillian Term	Quantity	Value(s)
Transmon frequency	$\omega_q \|e\rangle\langle e\|$	$\omega_q/(2\pi)$	4.97 GHz
Storage cavity frequencies	$\omega_m a_m^\dagger a_m$	$\omega_m/(2\pi)$	5.716, 5.965, 6.223, 6.469 GHz
Readout frequency	$\omega_r a_r^\dagger a_r$	$\omega_r/(2\pi)$	7.79 GHz
Readout dispersive shift	$\chi_r a_r^\dagger a_r \|e\rangle\langle e\|$	$\chi_r/(2\pi)$	1 MHz
Storage mode dispersive shifts	$\chi_m a_m^\dagger a_m \|e\rangle\langle e\|$	$\chi_m/(2\pi)$	-1.636, -1.269, -1.093, -0.906 MHz
Storage mode self-Kerrs	$\frac{k_m}{2} a_m^\dagger a_m (a_m^\dagger a_m - 1)$	$k_m/(2\pi)$	9.0, 5.2, 4.2, 3.2 kHz
Storage mode cross-Kerrs	$k_{mn} a_m^\dagger a_m a_n^\dagger a_n$	$k_{mn}/(2\pi)$	$-6 - 0$ kHz
Transmon $\|e\rangle \to \|g\rangle$ relaxation	$\frac{1}{T_1^q}(1+\bar{n})\mathcal{D}\big[\|g\rangle\langle e\|\big]$	T_1^q	108 ± 7 μs
Transmon $\|g\rangle - \|e\rangle$ dephasing	$\big(\frac{1}{T_2^q} - \frac{1}{2T_1^q}\big)\mathcal{D}\big[\|e\rangle\langle e\|\big]$	T_2^q	165 ± 14 μs
Readout linewidth	$\kappa_r \mathcal{D}[a_r]$	$\kappa_r/(2\pi)$	0.52 MHz
Storage mode relaxation	$\frac{1}{T_1^m}\mathcal{D}[a]$	T_1^m	$\sim 1 - 2$ ms, see [2]
Transmon thermal population	$\frac{\bar{n}}{T_1^q}\mathcal{D}\big[\|e\rangle\langle g\|\big]$	$\bar{n}$	1.5 ± 0.5 %
Storage mode dephasing	$\big(\frac{1}{T_2^m} - \frac{1}{2T_1^m}\big)\mathcal{D}\big[\|1\rangle\langle 1\|\big]$	T_2^m	$\sim 1.5 - 3$ ms, see [2]

Supplementary Table 1 | Multimode cQED system parameters

where $p_{W^2}(\vec{\boldsymbol{\alpha}}) = C_M|\tilde{W}_\sigma(\vec{\boldsymbol{\alpha}},\vec{\boldsymbol{\theta}})|^2$ satisfies $p_{W^2}(\vec{\boldsymbol{\alpha}}) \geq 0$ and $\int \mathrm{d}^{2M}\vec{\boldsymbol{\alpha}}\ p_{W^2}(\vec{\boldsymbol{\alpha}}) = \mathrm{Tr}[\sigma^2] = 1$, such that we can treat $p_{W^2}(\vec{\boldsymbol{\alpha}})$ as a probability distribution function that we can then use to sample a set of displacement vectors $\{\vec{\boldsymbol{\alpha}}^{(k)}\}$. For physical ρ and $\vec{\boldsymbol{\theta}}$, explicitly taking the real part is not necessary (as the integral will have 0 imaginary part, but we explicitly take the real part to emphasize that $F(\rho,\sigma)$ is a real quantity. Given a set $\{\vec{\boldsymbol{\alpha}}^{(k)}\}$, we can measure $\mathrm{Re}[e^{i\phi(\vec{\boldsymbol{\alpha}}^{(k)})}\tilde{W}_\rho(\vec{\boldsymbol{\alpha}}^{(k)},-\vec{\boldsymbol{\theta}})]$ and calculate the average $\frac{\mathrm{Re}[e^{i\phi(\vec{\boldsymbol{\alpha}}^{(k)})}\tilde{W}_\rho(\vec{\boldsymbol{\alpha}}^{(k)},-\vec{\boldsymbol{\theta}})]}{|\tilde{W}_\sigma(\vec{\boldsymbol{\alpha}}^{(k)},-\vec{\boldsymbol{\theta}})|}$ to obtain an estimate of $F(\rho,\sigma)$. In Supplementary Note 5, we show explicitly that by repeatedly measuring whether the qubit is in the $|g\rangle$ level, we can obtain a series of binomial outcomes $A_j^{(k)} \in \{1,-1\}$ whose expectation values lead to exactly $\mathrm{Re}[e^{i\phi(\vec{\boldsymbol{\alpha}}^{(k)})}\tilde{W}_\rho(\vec{\boldsymbol{\alpha}}^{(k)},-\vec{\boldsymbol{\theta}})]$. We call the above sampling method the W^2 tomography sampling method.

In general, we have the freedom to choose other probability distribution functions $p(\vec{\boldsymbol{\alpha}})$ to generate sampling points $\{\vec{\boldsymbol{\alpha}}^{(k)}\}$, since

$$\begin{aligned}&\mathrm{Tr}[\rho O]\\&= C_M \int \mathrm{d}^{2M}\vec{\boldsymbol{\alpha}}\ p(\vec{\boldsymbol{\alpha}})\frac{|\tilde{W}_O(\vec{\boldsymbol{\alpha}},\vec{\boldsymbol{\theta}})|}{p(\vec{\boldsymbol{\alpha}})}\mathrm{Re}[e^{i\phi(\vec{\boldsymbol{\alpha}})}\tilde{W}_\rho(\vec{\boldsymbol{\alpha}},-\vec{\boldsymbol{\theta}})].\end{aligned} \tag{9}$$

We can calculate the average of $C_M\frac{|\tilde{W}_O(\vec{\boldsymbol{\alpha}}^{(k)},\vec{\boldsymbol{\theta}})|}{p(\vec{\boldsymbol{\alpha}}^{(k)})}A_j^{(k)}$ to obtain an estimate for $\mathrm{Tr}[\rho O]$. However, in certain situations, there will be an optimal choice for $p(\vec{\boldsymbol{\alpha}})$. For example, if we perform single shot measurements where we only measure each sampling vector $\vec{\boldsymbol{\alpha}}^{(k)}$ once, the variance of the estimator $C_M\frac{|\tilde{W}_O(\vec{\boldsymbol{\alpha}}^{(k)},\vec{\boldsymbol{\theta}})|}{p(\vec{\boldsymbol{\alpha}}^{(k)})}A^{(k)}$ will be limited by

$$\begin{aligned}&C_M^2\int \mathrm{d}^{2M}\vec{\boldsymbol{\alpha}}\ p(\vec{\boldsymbol{\alpha}})\frac{|\tilde{W}_O(\vec{\boldsymbol{\alpha}},\vec{\boldsymbol{\theta}})|^2}{p^2(\vec{\boldsymbol{\alpha}})} - (\mathrm{Tr}[\rho O])^2\\&\geq \frac{[C_M\int \mathrm{d}^{2M}\vec{\boldsymbol{\alpha}}\ |\tilde{W}_O(\vec{\boldsymbol{\alpha}},\vec{\boldsymbol{\theta}})|]^2}{\int \mathrm{d}^{2M}\vec{\boldsymbol{\alpha}}\ p(\vec{\boldsymbol{\alpha}})} - (\mathrm{Tr}[\rho O])^2\\&= \left[C_M\int \mathrm{d}^{2M}\vec{\boldsymbol{\alpha}}\ |\tilde{W}_O(\vec{\boldsymbol{\alpha}},\vec{\boldsymbol{\theta}})|\right]^2 - (\mathrm{Tr}[\rho O])^2.\end{aligned} \tag{10}$$

Here, we have used the Cauchy-Schwarz inequality and the fact that $\int \mathrm{d}^{2M}\vec{\boldsymbol{\alpha}}\ p(\vec{\boldsymbol{\alpha}}) = 1$. The minimum is achieved when $p(\vec{\boldsymbol{\alpha}}) \propto |\tilde{W}_O(\vec{\boldsymbol{\alpha}},\vec{\boldsymbol{\theta}})|$, which we call the DEMESST sampling method. It is also worth noting that, for the W^2 method where $p_{W^2}(\vec{\boldsymbol{\alpha}}) = C_M|\tilde{W}_O(\vec{\boldsymbol{\alpha}},\vec{\boldsymbol{\theta}})|^2$, the integral shown in the first line of Supplementary Eqn. (10) will be divergent. To avoid this, Ref. [5, 6] have proposed choosing a threshold cutoff value to discard some sampling vectors $\vec{\boldsymbol{\alpha}}^{(k)}$ that make the denominator of $\frac{A_j^{(k)}}{|\tilde{W}_O(\vec{\boldsymbol{\alpha}}^{(k)},\vec{\boldsymbol{\theta}})|}$ too small. This cutoff procedure can lead to bias when estimating $\mathrm{Tr}[\rho O]$ and makes the error analysis more complicated. A detailed analysis of the effect of this cutoff in a multimode setting is beyond the scope of this work.

Instead, we focus on the DEMESST method. The probability distribution function is given by

$$p_{\mathrm{D}}(\vec{\boldsymbol{\alpha}}) = \frac{|\tilde{W}_O(\vec{\boldsymbol{\alpha}},\vec{\boldsymbol{\theta}})|}{Z_O}, \tag{11}$$

where

$$Z_O = \int \mathrm{d}^{2M}\vec{\boldsymbol{\alpha}}\ |\tilde{W}_O(\vec{\boldsymbol{\alpha}},\vec{\boldsymbol{\theta}})|. \tag{12}$$

In the DEMESST method, we must average $C_M Z_O A_j^{(k)}$ over all sampling vectors $\vec{\boldsymbol{\alpha}}^{(k)}$ and all possible binomial

outcomes from the qubit measurement per sampling vector. In the limit where we do one qubit measurement per $\vec{\boldsymbol{\alpha}}^{(k)}$, we can use Hoeffding's inequality to estimate the number of samples $N_{\rm spl}$ required to reach an accuracy ϵ_1 with probability $1-\delta_1$ to be

$$P\left(\left|\frac{C_M Z_O}{N_{\rm spl}}\sum_{k=1}^{N_{\rm spl}} A^{(k)} - \mathrm{Tr}[\rho O]\right| \geq \epsilon_1\right) \leq \delta_1 \tag{13}$$

when

$$N_{\rm spl} \geq \lceil \frac{2C_M^2 Z_O^2}{\epsilon_1^2}\ln(2/\delta_1)\rceil. \tag{14}$$

We can see that, in general, $N_{\rm spl} \propto (C_M Z_O)^2$. In the next note, we analyze the properties of Z_O for our operators of interest.

Supplementary Note 4 – Density matrix reconstruction procedure

In this note, we discuss our scheme for reconstructing an unknown state using the DEMESST sampling method to estimate each element of its density operator in the Fock basis. We consider a system with M modes and maximum total photon number N between those modes. This restricts the dimension of the Hilbert space to $\binom{M+N}{N}$. We focus on the scaling of the sampling overhead (number of samples required) vs. mode number M, in the limit where M is much larger than $2N$, and show that this overhead scales polynomially vs. M with bounded photon number N, demonstrating the efficiency of the DEMESST approach as M increases.

We assume that the operator O (in $\mathrm{Tr}[\rho O]$) is in one of the following forms:

$$\begin{aligned} O_{\vec{\mathbf{n}},\vec{\mathbf{n}}} &= |\vec{\mathbf{n}}\rangle\langle\vec{\mathbf{n}}|,\\ O^R_{\vec{\mathbf{n}},\vec{\mathbf{n}}'} &= \frac{|\vec{\mathbf{n}}\rangle\langle\vec{\mathbf{n}}'| + |\vec{\mathbf{n}}'\rangle\langle\vec{\mathbf{n}}|}{\sqrt{2}} \quad (\vec{\mathbf{n}} \neq \vec{\mathbf{n}}'),\\ O^I_{\vec{\mathbf{n}},\vec{\mathbf{n}}'} &= i\frac{|\vec{\mathbf{n}}\rangle\langle\vec{\mathbf{n}}'| - |\vec{\mathbf{n}}'\rangle\langle\vec{\mathbf{n}}|}{\sqrt{2}} \quad (\vec{\mathbf{n}} \neq \vec{\mathbf{n}}'). \end{aligned} \tag{15}$$

Here $|\vec{\mathbf{n}}\rangle = \bigotimes_{m=1}^M |n_m\rangle$ and $|\vec{\mathbf{n}}'\rangle = \bigotimes_{m=1}^M |n'_m\rangle$ are Fock basis states that satisfy $\sum_{m=1}^M n_m \leq N$ and $\sum_{m=1}^M n'_m \leq N$. The operators O are chosen such that $O = O^\dagger$ and $\mathrm{Tr}[O^\dagger O] = 1$. For a system with M modes and maximum total photon number N, we have $\binom{M+N}{N}^2$ of these operators.

One essential observation is that, when $M > 2N$, for any $(\vec{\mathbf{n}}, \vec{\mathbf{n}}')$ pair there are at least $(M-2N)$ elements in the set $S \equiv S_{(\vec{\mathbf{n}},\vec{\mathbf{n}}')} = \{m | n_m = n'_m = 0\}$. We also denote $\bar{S} = \{1, 2, \ldots, M\} \backslash S$. Because of this, the corresponding operators shown in Supplementary Eqn. (15) can always be decomposed as

$$O = \Big(\bigotimes_{m\in S} |0\rangle\langle 0|_m\Big) \otimes O_{\bar{S}}, \tag{16}$$

where $O_{\bar{S}}$ has support on the modes with index $m \in \bar{S}$. We can see that the number of elements in $\bar{S}$ is no greater than $2N$, and independent of M. Similarly, the generalized Wigner function of such an operator O satisfies

$$\begin{aligned} \tilde{W}_O(\vec{\boldsymbol{\alpha}}, \vec{\boldsymbol{\theta}}) = \Big(\prod_{m\in S} &\tilde{W}_{|0\rangle\langle 0|}(\alpha_m, \theta_m)\Big)\\ &\cdot \tilde{W}_{O_{\bar{S}}}(\vec{\boldsymbol{\alpha}}_{\bar{S}}, \vec{\boldsymbol{\theta}}_{\bar{S}}), \end{aligned} \tag{17}$$

where $\vec{\boldsymbol{\alpha}}_{\bar{S}}$, $\vec{\boldsymbol{\theta}}_{\bar{S}}$ contain those elements in $\vec{\boldsymbol{\alpha}}$, $\vec{\boldsymbol{\theta}}$ whose mode index $m \in \bar{S}$. Again, $\tilde{W}_{O_{\bar{S}}}(\vec{\boldsymbol{\alpha}}_{\bar{S}}, \vec{\boldsymbol{\theta}}_{\bar{S}})$ is independent of M.

Now, we consider the sampling overhead to obtain a precise estimate of $\mathrm{Tr}[\rho O]$ when O satisfies the properties described above. One approach is to sample directly according to the M-mode function $|\tilde{W}_O(\vec{\boldsymbol{\alpha}}, \vec{\boldsymbol{\theta}})|/Z_O$. Based on Supplementary Eqn. (14), the key quantity we should focus on is $C_M Z_O$, which satisfies

$$\begin{aligned} C_M Z_O &= \Big(\prod_{m=1}^M \frac{2(1-\cos\theta_m)}{\pi}\Big)\int \mathrm{d}^{2M}\vec{\boldsymbol{\alpha}}\ |\tilde{W}_O(\vec{\boldsymbol{\alpha}}, \vec{\boldsymbol{\theta}})|\\ &= \prod_{m\in S}\Big(\frac{2(1-\cos\theta_m)}{\pi}\int \mathrm{d}^2\vec{\boldsymbol{\alpha}}\ |\tilde{W}_{|0\rangle\langle 0|}(\alpha_m, \theta_m)|\Big)\\ &\quad \cdot C_{\bar{S}}\int \mathrm{d}^{2|\bar{S}|}\vec{\boldsymbol{\alpha}}_{\bar{S}}\ |\tilde{W}_{O_{\bar{S}}}(\vec{\boldsymbol{\alpha}}_{\bar{S}}, \vec{\boldsymbol{\theta}}_{\bar{S}})|\\ &= 2^{M-|\bar{S}|} C_{\bar{S}} Z_{O_{\bar{S}}}. \end{aligned} \tag{18}$$

Here, $C_{\bar{S}} = \prod_{m\in\bar{S}}[2(1-\cos\theta_m)/\pi]$. Since $O_{\bar{S}}$ is supported on at most $2N$ modes, which is independent of M when $M > 2N$, the only M-dependence in $C_M Z_O$ comes from the 2^M factor. Unfortunately, this is still unfavorable, as it grows exponentially with mode number M.

To resolve this issue, consider $O_{\bar{S}}$, which is nontrivially supported on the modes contained in $\bar{S}$, rather than the full operator O itself. We introduce the projection operator $P_S = \bigotimes_{m\in S}|0\rangle\langle 0|_m$ and denote $\rho_{\bar{S}} = \mathrm{Tr}_S[\rho P_S]$. Here $\mathrm{Tr}_S[\bullet]$ means the partial trace over all modes with $m \in S$. We can see that

$$\mathrm{Tr}[\rho O] = \mathrm{Tr}[\rho_{\bar{S}} O_{\bar{S}}], \tag{19}$$

where $\rho_{\bar{S}}$ and $O_{\bar{S}}$ are wholly supported on modes in $\bar{S}$, which contains at most $2N$ elements. We can perform DEMESST sampling according to $O_{\bar{S}}$ as follows:

$$\begin{aligned} &\mathrm{Tr}[\rho_{\bar{S}} O_{\bar{S}}]\\ &= C_{\bar{S}} Z_{O_{\bar{S}}}\int \mathrm{d}^{2|\bar{S}|}\vec{\boldsymbol{\alpha}}_{\bar{S}}\ p_{\rm D}(\vec{\boldsymbol{\alpha}}_{\bar{S}})\ \mathrm{Re}[e^{i\phi(\vec{\boldsymbol{\alpha}}_{\bar{S}})}\tilde{W}_{\rho_{\bar{S}}}(\vec{\boldsymbol{\alpha}}_{\bar{S}}, -\vec{\boldsymbol{\theta}}_{\bar{S}})], \end{aligned} \tag{20}$$

where p_D is the probability distribution function defined in Supplementary Eqn. (11). In the experiment, we utilize the $|f\rangle$ level of the transmon to effectively restrict the cavity state to $\rho_{\bar{S}}$. From the measurement, we obtain binomial outcomes $A_j^{(k)} \in \{1, -1\}$ with expectation values

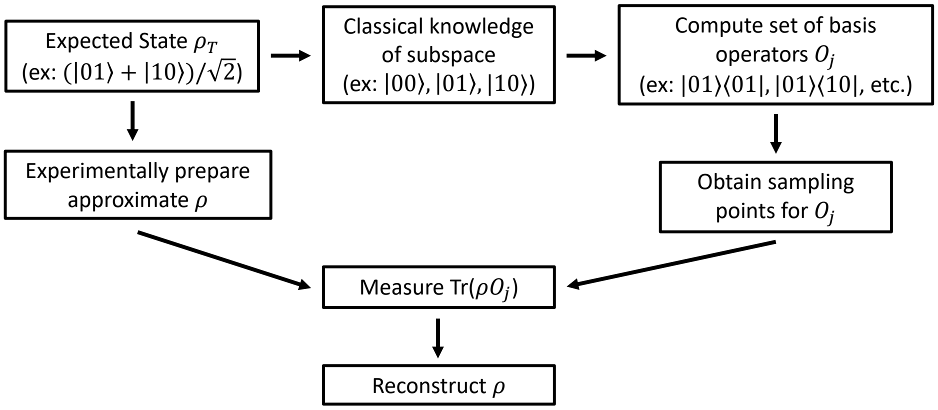

Supplementary Figure 1 | Flowchart depicting the steps involved in our density matrix reconstruction procedure, of which DEMESST is an example. Starting with a target expected state ρ_T, we can use our knowledge of ρ_T to identify a target subspace and compute the operators O_j that span that basis. We then use those operators to generate sampling points, which may involve projections to be done efficiently, as described in Supplementary Note 3. By experimentally implementing the operators on our prepared state, we can reconstruct the state ρ and compare it with the ideal target ρ_T. Even if the prepared state ρ is not close to ρ_T, as long as it lives in the same subspace, we can effectively reconstruct it.

$\mathrm{Re}[e^{i\phi(\vec{\boldsymbol{\alpha}}_{\bar{S}}^{(k)})}\tilde{W}_{\rho_{\bar{S}}}(\vec{\boldsymbol{\alpha}}_{\bar{S}}^{(k)}, -\vec{\boldsymbol{\theta}}_{\bar{S}})]$. More details about this experimental protocol are presented in Supplementary Note 5. Like before, we can use Hoeffding's inequality to estimate the sampling overhead. If we only measure once per sampling vector $\vec{\boldsymbol{\alpha}}_{\bar{S}}^{(k)}$, we will have

$$P\left(\left|\frac{C_{\bar{S}}Z_{O_{\bar{S}}}}{N_{\mathrm{spl}}}\sum_{k=1}^{N_{\mathrm{spl}}}A^{(k)} - \mathrm{Tr}[\rho_{\bar{S}}O_{\bar{S}}]\right| \geq \epsilon_2\right) \leq \delta_2 \quad (21)$$

when

$$N_{\mathrm{spl}} \geq \lceil \frac{2C_{\bar{S}}^2 Z_{O_{\bar{S}}}^2}{\epsilon_2^2}\ln(2/\delta_2)\rceil. \quad (22)$$

When N is bounded, $C_{\bar{S}}Z_{O_{\bar{S}}}$ is independent of mode number M when $M > 2N$. Therefore, N_{spl} in Supplementary Eqn. (22) scales as

$$N_{\mathrm{spl}} \sim \mathcal{O}_M\Big(\frac{f(N)}{\epsilon_2^2}\ln(2/\delta_2)\Big), \quad (23)$$

where $\mathcal{O}_M$ indicates that we focus only on the scaling over M in the large M limit, and $f(N) = 2C_{\bar{S}}^2 Z_{O_{\bar{S}}}^2$ is a function that depend solely on N and the specific form of O from Supplementary Eqn. (15). We also introduce $f_{\max}(N)$ to represent the maximum value of $f(N)$ from those $\binom{M+N}{N}^2$ operators.

We can now consider our reconstructed density matrix $\hat{\rho}$. By performing expectation value estimation on the unknown state ρ with all $\binom{M+N}{N}^2$ operators with form in Supplementary Eqn. (15), we can achieve

$$P(B) \geq 1 - \binom{M+N}{N}^2\delta_2 \quad (24)$$

with total sample number

$$N_{\mathrm{tot}} \sim \mathcal{O}_M\left[\binom{M+N}{N}^2\frac{f_{\max}(N)}{\epsilon_2^2}\ln(2/\delta_2)\right], \quad (25)$$

where B requires all the conditions below:

$$\begin{aligned}&|\langle\vec{\mathbf{n}}|(\hat{\rho}-\rho)|\vec{\mathbf{n}}\rangle| \leq \epsilon_2,\\ &|\mathrm{Re}[\langle\vec{\mathbf{n}}|(\hat{\rho}-\rho)|\vec{\mathbf{n}}'\rangle]| \leq \epsilon_2/\sqrt{2} \quad (\vec{\mathbf{n}} \neq \vec{\mathbf{n}}'),\\ &|\mathrm{Im}[\langle\vec{\mathbf{n}}|(\hat{\rho}-\rho)|\vec{\mathbf{n}}'\rangle]| \leq \epsilon_2/\sqrt{2} \quad (\vec{\mathbf{n}} \neq \vec{\mathbf{n}}').\end{aligned} \quad (26)$$

If B is satisfied, we will have the Frobenius norm distance between our reconstructed state and the true state satisfy

$$||\hat{\rho}-\rho||_F = \sqrt{\sum_{\vec{\mathbf{n}},\vec{\mathbf{n}}'}|\langle\vec{\mathbf{n}}|(\hat{\rho}-\rho)|\vec{\mathbf{n}}'\rangle|^2} \leq \binom{M+N}{N}\epsilon_2. \quad (27)$$

Also, if ρ is a pure state, we will have

$$\begin{aligned}|F(\hat{\rho},\rho)-1| &= |\mathrm{Tr}[(\hat{\rho}-\rho)\rho]| \leq \epsilon_2\cdot\sum_{\vec{\mathbf{n}},\vec{\mathbf{n}}'}|\langle n|\rho|\vec{\mathbf{n}}'\rangle|\\ &\leq \epsilon_2\cdot\sqrt{\sum_{\vec{\mathbf{n}},\vec{\mathbf{n}}'}|\langle n|\rho|\vec{\mathbf{n}}'\rangle|^2}\cdot\binom{M+N}{N}\\ &= \epsilon_2\cdot\sqrt{\mathrm{Tr}[\rho^2]}\cdot\binom{M+N}{N} = \binom{M+N}{N}\epsilon_2.\end{aligned} \quad (28)$$

In summary, by choosing $\epsilon_2 = \epsilon/\binom{M+N}{N}$ and $\delta_2 = \delta/\binom{M+N}{N}^2$, our sampling method will require the total sample number

$$N_{\mathrm{tot}} \sim \mathcal{O}_M\left[\binom{M+N}{N}^4\frac{f_{\max}(N)}{\epsilon^2}\ln\left(2\binom{M+N}{N}^2/\delta\right)\right] \quad (29)$$

to achieve

$$P(||\hat{\rho} - \rho||_F \leq \epsilon) \geq 1 - \delta, \tag{30}$$

even if we only perform a single measurement for each sampling instance. With the same amount of sampling, we can also achieve

$$P(|F(\hat{\rho}, \rho) - 1| \leq \epsilon) \geq 1 - \delta \tag{31}$$

when ρ is a pure state. We note that in the above, we consider a fixed N and focus on the scaling over M. The source of the sampling overhead's N dependence is the increased subspace dimension and number of O operators. However, the subject of our work is the protocol's dependence on M.

The procedure for DEMESST is as follows: first, the Wigner function corresponding to a chosen basis operator is normalized to a probability distribution based on its absolute value. Then, displacement vectors are sampled from the resulting inverse cumulative distribution function (CDF) by randomly selecting a value between 0 and 1 to find the corresponding angular and radial values of the displacements. This calculation is performed efficiently by utilizing Laguerre functions and their inverses. During measurement, each displacement vector will have the original sign of its Wigner function value preserved, so that if the Wigner function was negative at that point, the final measured value will be multiplied by -1. Observing a set of these will provide an estimate of the chosen density matrix element. Repeating this for multiple elements will thus produce the density matrix of the prepared state.

Supplementary Note 5 – DEMESST Experimental Protocol

For simplicity, in this note we assume that the cavity is initialized as a pure state $|\psi\rangle$. However, the same arguments will apply for a generic density operator ρ, which can always be decomposed as $\rho = \sum_i c_i|\psi_i\rangle\langle\psi_i|$ and understood as an ensemble average of a set of pure states $\{|\psi_i\rangle\langle\psi_i|\}$ with probability c_i.

First, we consider the generalized Wigner function of an M-mode state. We assume that the cavity-qubit state is initialized as $|\psi\rangle|g\rangle$. To perform the Wigner tomography measurement, we apply a short (large-bandwidth) drive to each cavity mode, then apply a large-bandwidth $\pi/2$ pulse on the qubit to begin the parity measurement. After these operations, the qubit part becomes $\exp(-i\frac{\pi}{4}\sigma_y)|g\rangle = \frac{|g\rangle+|e\rangle}{\sqrt{2}}$, and the cavity part becomes $|\psi_D\rangle = D(-\vec{\boldsymbol{\alpha}})|\psi\rangle$. Then, as part of the parity measurement, we wait for a time t. Due to the dispersive interaction Hamiltonian $H_{\text{int}} = \sum_m \chi_m a_m^\dagger a_m |e\rangle\langle e|$, the cavity modes will be entangled with the qubit as $\frac{1}{\sqrt{2}}[|\psi_D\rangle|g\rangle + e^{-i\sum_m \theta_m a_m^\dagger a_m}|\psi_D\rangle|e\rangle]$, where $\theta_m = \chi_m t$. In principle, we could choose any time t, as long as none of the θ_m are integer multiples of 2π. However, in practice, we select t to make each of the θ_m as close to π modulo 2π as possible. This choice provides the maximum contrast and is closest to the ideal multimode parity operator. To complete our generalized parity measurement, we apply another $\pi/2$ pulse on the qubit, but with different phase from the initial one. Specifically, we consider the qubit rotation along $\vec{r} = -\sin\phi\,\vec{e}_x - \cos\phi\,\vec{e}_y$. By applying this $\exp(-i\frac{\pi}{4}\vec{r}\cdot\vec{\sigma})$ operation, the final cavity-qubit entangled state $|\Psi\rangle$ will be

$$\begin{aligned}|\Psi\rangle = &\frac{|\psi_D\rangle + e^{i\phi}e^{-i\sum_m \theta_m a_m^\dagger a_m}|\psi_D\rangle}{2}|g\rangle \\ &+ \frac{-e^{-i\phi}|\psi_D\rangle + e^{-i\sum_m \theta_m a_m^\dagger a_m}|\psi_D\rangle}{2}|e\rangle.\end{aligned} \tag{32}$$

Thus, when performing readout on the qubit, the final probability of achieving $|g\rangle$ will be

$$\begin{aligned}P_g &= \frac{1 + \text{Re}\{e^{i\phi}\text{Tr}[D^\dagger(\vec{\alpha})|\psi\rangle\langle\psi|D(\vec{\alpha})e^{-i\sum_m \theta_m a_m^\dagger a_m}]\}}{2} \\ &= \frac{1}{2}\{1 + \text{Re}[e^{i\phi}\tilde{W}_{|\psi\rangle\langle\psi|}(\vec{\alpha}, -\vec{\boldsymbol{\theta}})]\}.\end{aligned} \tag{33}$$

Therefore, if we record $A = 1$ upon measuring $|g\rangle$ and $A = -1$ otherwise, the expectation value of A will be exactly $\text{Re}[e^{i\phi}\tilde{W}_\rho(\vec{\alpha}, -\vec{\boldsymbol{\theta}})]$. This derivation applies for any ϕ, but as mentioned before, the choice of ϕ depends on the operator O and the sampling vector $\vec{\alpha}$.

We must modify the experimental protocol above to measure the generalized Wigner function for the projected state $\rho_{\bar{S}}$, which is defined in Supplementary Note 4. In particular, we utilize the second excited state $|f\rangle$ of the transmon to implement subsystem tomography and measure the Wigner values for only the projected states $\rho_{\bar{S}}$, which is similar to the idea used in [7]. Our W states are generated using multimode photon blockade as described in [1] to ensure that our maximum total photon number is $N = 1$. Consequently, for the density matrix reconstruction, the Hilbert space will be spanned by $\{|\vec{\mathbf{n}}\rangle \mid \sum_{m=1}^M n_m \leq 1\}$. In this case, the projected operator $O_{\bar{S}}$ introduced in Supplementary Eqn. (16) will be supported on at most 2 modes. Because of the different dispersive couplings between the qubit and distinct cavity modes (STable 1), we can selectively target each of the modes with sufficiently narrow-bandwidth qubit pulses to help perform the necessary projections. Therefore, before the parity measurement, we first apply several long (narrow-bandwidth) qubit π pulses with frequencies $\omega_q + \chi_m$ for $m \in S$, such that the qubit that coupled with the $(I - P_S)|\psi\rangle$ component of the multimode cavity state will transfer from $|g\rangle$ to $|e\rangle$, while the component that coupled with $P_S|\psi\rangle$ will stay in $|g\rangle$. Then we give the transmon a short π pulse on the $|e\rangle - |f\rangle$ transition. After those steps, the cavity-transmon state becomes

$$|\Psi\rangle = P_S|\psi\rangle|g\rangle + (I - P_S)|\psi\rangle|f\rangle. \tag{34}$$

Finally, we can use the procedure that we described before when focusing on the Wigner value measurement of a generic M-mode state ρ. We only need to drive those modes with index $m \in \bar{S}$ such that those modes are displaced by $D(-\vec{\alpha}_{\bar{S}})$. The probability to measure $|g\rangle$ from the final qubit readout is

$$P_{g,\phi} = \frac{\mathrm{Tr}[\rho_{\bar{S}}] + \mathrm{Re}[e^{i\phi}\tilde{W}_{\rho_{\bar{S}}}(\vec{\alpha}_{\bar{S}}, -\vec{\boldsymbol{\theta}}_{\bar{S}})]}{2}, \quad (35)$$

where $\rho_{\bar{S}} = \mathrm{Tr}_S[P_S|\psi\rangle\langle\psi|]$. In practice, the result above is unaffected by the order in which we perform the qubit π pulses and cavity displacements, assuming that we add additional π pulses to target the displaced state. We found this order to work better in the experiment, despite the need for a larger comb of π pulse frequencies.

Now, if we proceed similarly to before and assign $A = 1$ for $|g\rangle$ and $A = -1$ otherwise, the expectation value of A will not give us our desired result. To solve this issue, we utilize the freedom we have in choosing the phase of the second qubit $\pi/2$ pulse in the parity measurement. If we choose the second qubit $\pi/2$ rotation to be along $-\vec{r}$ instead of $\vec{r}$ (or equivalently choose $(\phi+\pi)$ instead of ϕ, then the probability of measuring $|g\rangle$ will be

$$P_{g,(\phi+\pi)} = \frac{\mathrm{Tr}[\rho_{\bar{S}}] - \mathrm{Re}[e^{i\phi}\tilde{W}_{\rho_{\bar{S}}}(\vec{\alpha}_{\bar{S}}, -\vec{\boldsymbol{\theta}}_{\bar{S}})]}{2}. \quad (36)$$

Therefore, comparing with Supplementary Eqn. (35), one solution to recover our desired quantity is to first generate a random binomial number s. There is a 50% probability that $s = 1$ and 50% probability that $s = -1$. If we get $s = 1$, we choose the second qubit $\pi/2$ rotation to be along $\vec{r}$, and otherwise choose $-\vec{r}$ instead. In both cases, we assign $A = 1$ if the qubit measurement outcome is $|g\rangle$, and assign $A = -1$ if it is not $|g\rangle$. The expectation value of sA will be exactly $\mathrm{Re}[e^{i\phi}\tilde{W}_{\rho_{\bar{S}}}(\vec{\alpha}_{\bar{S}}, -\vec{\boldsymbol{\theta}}_{\bar{S}})]$. An advantage of this procedure is that it will work even when it is difficult to distinguish $|e\rangle$ and $|f\rangle$ levels in qubit readout, as long as we can distinguish $|g\rangle$ outcomes from others. The same applies for other permutations of these three states, as long as the experimental protocol is adjusted accordingly. In the actual experiment, we did not use this trick of random number s generation, since we can perform more than a single measurement per sampling vector $\vec{\alpha}_{\bar{S}}$. Instead, we repeated the experiment 10 times for rotation of the second $\pi/2$ along $\vec{r}$ and 10 times along $-\vec{r}$. Finally, we subtracted the averaged probability of measuring $|g\rangle$ between the two cases to obtain an estimate for $\mathrm{Re}[e^{i\phi}\tilde{W}_{\rho_{\bar{S}}}(\vec{\alpha}_{\bar{S}}, -\vec{\boldsymbol{\theta}}_{\bar{S}})]$.

For the data collected in the main text, the transmon readout fidelity was $\approx 80\%$. The error associated with this fidelity is defined as the overlap between $|g\rangle$ and $|e\rangle$, and can be thought of as the sum of the probabilities of misidentifying $|g\rangle$ as $|e\rangle$ and vice versa. The transmon dephasing and relaxation times were not the limiting factor to this fidelity. Instead, it was because we used a relatively low-power readout tone. The lower power allowed us to decrease the magnitude of the cross-Kerr interaction between the readout cavity and the multimode storage cavity modes, which would cause readout errors by changing the readout frequency and thus its response.

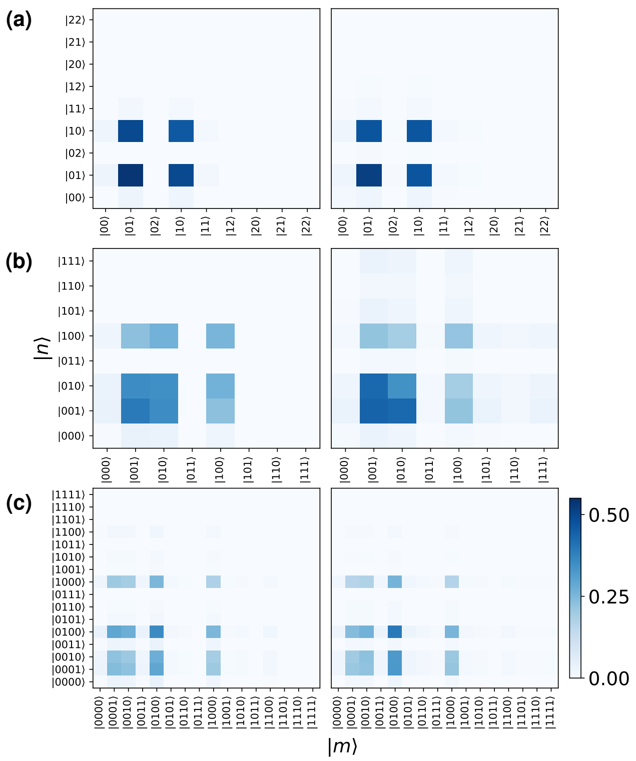

Supplementary Figure 2 | Absolute value of the final density matrices determined using the DEMESST tomography sampling method (left column) and the OLI method (right column) for W states of (a) 2, (b) 3, and (c) 4 modes. The results for DEMESST and OLI are in good agreement.

Supplementary Note 6 – Final Reconstructed Density Matrices

Using the DEMESST and OLI tomography methods, we can reconstruct the final density matrices of our prepared W states. These matrices are final in the sense that they are the results obtained from the entire set of measurements performed in the experiment, i.e. 100 averages for each distinct displacement, so that the total measurement number is 10 times the maximum measurement number shown in Fig. 3 of the main text. Following the discussion in Supplementary Note 3 about expectation value estimation with the DEMESST method, we consider the process where we sample N_{spl} displacement vectors $\vec{\boldsymbol{\alpha}}$ with probability density $p_D(\vec{\boldsymbol{\alpha}})$, and perform N_{ms} measurements for each $\vec{\boldsymbol{\alpha}}$ vector with binomial outcomes $A \in \{1, -1\}$ and expectation value $\mathrm{Re}[e^{i\phi(\vec{\boldsymbol{\alpha}})}\tilde{W}_{\rho}(\vec{\alpha}, -\vec{\boldsymbol{\theta}})]$ (denoted as $e(\vec{\boldsymbol{\alpha}})$ below). We find the variance of the

estimator $C_M Z_O \frac{\sum_{k=1}^{N_{\rm spl}} \sum_{j=1}^{N_{\rm ms}} A_j^{(k)}}{N_{\rm spl} N_{\rm ms}}$ to be:

$$\begin{aligned}\mathrm{Var} = &\frac{C_M^2 Z_O^2}{N_{\rm spl}}\Big[\int \mathrm{d}^{2M}\vec{\boldsymbol{\alpha}}\ p_D(\vec{\boldsymbol{\alpha}})e^2(\vec{\boldsymbol{\alpha}}) - \frac{(\mathrm{Tr}[\rho O])^2}{C_M^2 Z_O^2}\Big] \\ &+ \frac{C_M^2 Z_O^2}{N_{\rm spl} N_{\rm ms}}\Big[1 - \int \mathrm{d}^{2M}\vec{\boldsymbol{\alpha}}\ p_D(\vec{\boldsymbol{\alpha}})e^2(\vec{\boldsymbol{\alpha}})\Big].\end{aligned} \tag{37}$$

Since $\frac{\mathrm{Tr}[\rho O]}{C_M Z_O} = \int \mathrm{d}^{2M}\vec{\boldsymbol{\alpha}}\ p_D(\vec{\boldsymbol{\alpha}})e(\vec{\boldsymbol{\alpha}})$, the first line in Supplementary Eqn. (37) is no less than zero. Therefore, the choice of $N_{\rm ms} = 1$ will lead to the smallest variance when fixing the total number of measurements $N_{\rm tot} = N_{\rm spl} N_{\rm ms}$. In other words, given a fixed total measurement number that equals the product of a number of distinct sets of cavity displacements times the number of averages that each displacement is repeated, we would gain the most information from maximizing the number of distinct displacements and measuring each a single time. In practice, there are other considerations that lead us to instead choose to average each measurement 10 times (as mentioned in Supplementary Note 5, when using the $|f\rangle$ level projection protocol, we count 2 sequences with a π phase difference in the second $\frac{\pi}{2}$ pulses as one measurement). For one, the time required for our hardware to run more displacements was much longer than the time required for more averages. Keeping the total measurement number fixed while running twice as many displacements (half as many averages) would take roughly 1.8 times as long. Furthermore, we wanted to ensure that we obtained accurate measurement results despite our imperfect readout fidelity ($\approx 80\%$). Thus, we chose 10 averages to be a good middle ground for the required measurement time and sufficient to avoid miscalibration and biased expectation values. We then repeat this process 10 times to obtain statistics, resulting in a total of 100 repetitions for each distinct displacement. This choice lets us balance this theoretical maximal information of singleshot measurements with our measurement errors and measurement time.

The final reconstructed density matrices for 2, 3, and 4 modes are shown in Supplementary Fig. 2. We plot the absolute values of the density matrix elements so that we have a single matrix grid for each combination of tomography method and mode number. Additionally, to directly observe state preparation errors, for DEMESST we measure the multimode Fock basis elements with up to 2 photons, and for OLI we measure the full tensored $\{|0\rangle, |1\rangle\}^{\otimes M}$ space. We can see that the two methods are in good agreement, with the largest visible deviation being in the 3-mode case for Fock basis elements with nonzero population in the second (middle) cavity mode. Nevertheless, the distances between the two final matrices as determined by the two methods is still low, and is 0.05 for the 2-mode case, 0.22 for the 3-mode case, and 0.30 for the 4-mode case. These distances are all below the corresponding minimum distances at the maximum total measurement number presented in the main text, and so this difference should not have significantly affected those results.

Besides some slight deviations in the measured populations of individual density matrix elements, the remaining distance between the final reconstructed matrices can be explained by small differences in the fit phase angles of the W states. For the 2-mode W state with form $|W_2\rangle = (|10\rangle + e^{i\phi}|01\rangle)/\sqrt{2}$, using the DEMESST method we obtain a fit $\phi_D = 0.04$, and using the OLI method we measure $\phi_O = 0.03$. In the 3-mode case, for W state with phase angles defined as $|W_3\rangle = (|100\rangle + e^{i\phi_1}|010\rangle + e^{i\phi_2}|001\rangle)/\sqrt{3}$, we measure $\phi_{1D} = -0.19$ and $\phi_{2D} = 1.57$, while $\phi_{1O} = -0.12$ and $\phi_{2O} = 1.57$. Similarly to what we see in the populations, the deviation is primarily in the middle mode. Finally, for 4 modes and W state $|W_4\rangle = (|1000\rangle + e^{i\phi_1}|0100\rangle + e^{i\phi_2}|0010\rangle + e^{i\phi_3}|0001\rangle)/\sqrt{4}$, we find $\phi_{1D} = -1.36, \phi_{2D} = -2.90$, and $\phi_{3D} = 0.60$, and $\phi_{1O} = -1.38, \phi_{2O} = -3.02$, and $\phi_{3O} = 0.63$. These angles are obtained by discretely sweeping the ϕ_j values over the full 2π range for each of the modes and determining which set of ϕ_j gives the largest fidelity when compared to an ideal W state with those phases.

Supplementary Note 7 – Infidelity and Matrix Distance Simulations

In this note, we present simulations of the infidelity and Frobenius norm (F-norm) matrix distance vs. point number for our two tomography methods, DEMESST and OLI, following the same procedure described in the main text. We use the two methods to reconstruct an ideal W state $|W\rangle = \frac{1}{\sqrt{M}}\sum_{m=1}^{M}|1_m\rangle$, where $|1_m\rangle$ represents the multimode Fock state with a single photon in the m-th mode. For DEMESST, we simulate up to $M = 7$ modes, while for OLI we consider up to $M = 5$ modes. We vary the number of randomly chosen mode displacement vectors $\{\vec{\alpha}\}$, and observe the fidelity and the F-norm distance between the reconstructed density matrices and the ideal one, $|W\rangle\langle W|$. The results are shown in Supplementary Fig. 3 and Supplementary Fig. 4. For each set of randomly sampled $\vec{\boldsymbol{\alpha}}$ vectors, we perform the state reconstruction 96 times while modeling readout errors to produce the error bars.

We now discuss how to reconstruct the W state in a single run of the simulation. In DEMESST, we have $(M+1)^2$ basis operators (defined in Supplementary Eqn. (15)) for which we want to estimate expectation values. Following Supplementary Eqn. (19), we project $M-2$ modes out of the sampling problem. Then, we only need to sample 2-mode $\vec{\boldsymbol{\alpha}}_{\bar{S}}$ vectors and perform our generalized Wigner measurements in those remaining 2 modes. For each basis operator, we randomly sample 48–6144 $\vec{\boldsymbol{\alpha}}_{\bar{S}}$ vectors according to the probability density function $p_D(\vec{\boldsymbol{\alpha}}_{\bar{S}})$ (defined in Supplementary Eqn. (11)). For each $\vec{\boldsymbol{\alpha}}_{\bar{S}}$ vector, we simulate a generalized Wigner measurement with 10 averages. As explained in Sup-

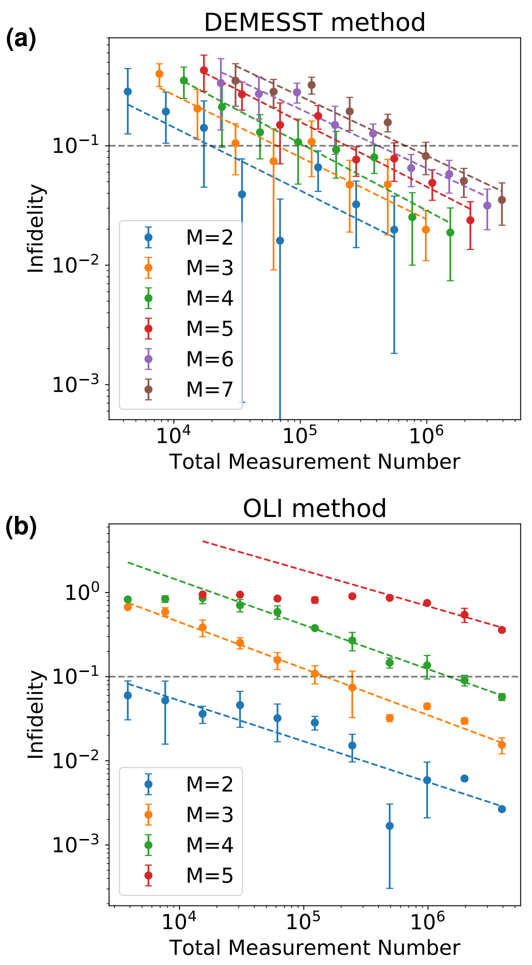

Supplementary Figure 3 | Simulated infidelity vs. total measurement number for W states of different mode numbers for the two tomography methods, (a) DEMESST and (b) OLI. The infidelity is computed as the fidelity difference between the reconstructed state at a given total measurement number vs. an ideal M-mode W state. Error bars are obtained from repeating the simulation multiple times while including readout errors, and indicate one standard deviation. The infidelities decrease to lower values more quickly for the DEMESST approach, especially for larger mode numbers. The dashed horizontal lines indicates an infidelity of 0.1 (90% fidelity).

plementary Note 5, we sample a binomial variable 10 times with probability $P_{g,\phi}$ to get the outcome $A = 1$, and another 10 times with probability $P_{g,(\phi+\pi)}$ to get the outcome $A = 1$ (see Supplementary Eqn. (35) and Supplementary Eqn. (36)). We use these results to estimate the expectation value for the basis operator. Repeating for all basis operators gives us the reconstructed density matrix. Finally, since it is possible for the reconstructed state to be not physical, we further utilize the algorithm introduced in Ref. [8] to find a maximally likely choice of density matrix that satisfies physicality constraints. We calculate the fidelity and F-norm distance of this physical density matrix with respect to the ideal $|W\rangle\langle W|$ state.

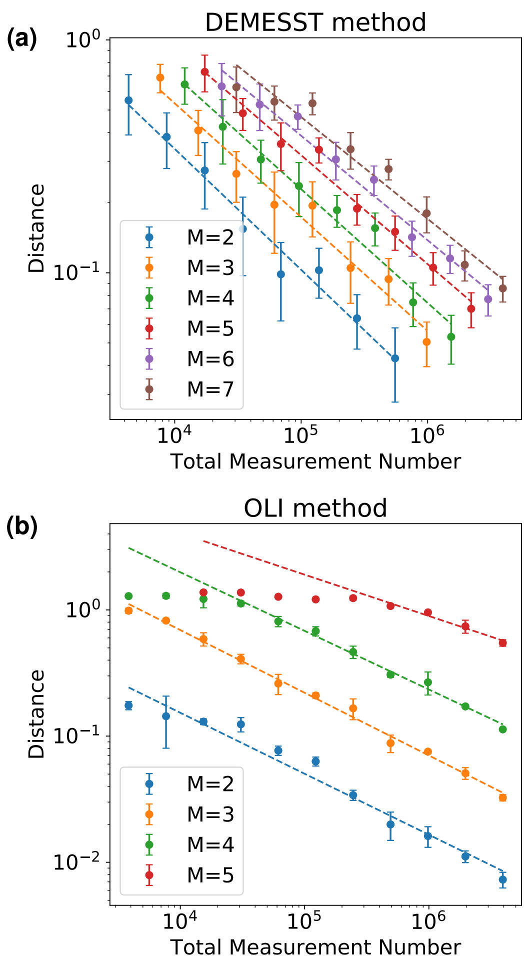

Supplementary Figure 4 | Simulated matrix distance vs. total measurement number for W states of different mode numbers for the two tomography methods, (a) DEMESST and (b) OLI. The distance is computed as the Frobenius norm between the reconstructed state at a given measurement number and an ideal M-mode W state. Error bars are obtained from repeating the simulation multiple times while including readout errors, and indicate one standard deviation. The distances decrease to lower values more quickly for the DEMESST approach, especially for larger mode numbers.

The results are shown in Supplementary Fig. 3(a) and 4(a). The Total Measurement Number is $(M+1)^2$ basis operators, times the number of sampled $\vec{\boldsymbol{\alpha}}_{\bar{S}}$ vectors per basis operator, times 10 measurements per $\vec{\boldsymbol{\alpha}}_{\bar{S}}$ vector.

In practice, we have imperfect qubit readout. We model this in the simulation in the following way: if the binomial outcome is $A = 1$ (qubit in $|g\rangle$), the readout voltage V is generated by a normal distribution with mean V_g and variance σ_g^2. If instead $A = -1$ (qubit in $|e\rangle$ or $|f\rangle$), V is generated by a normal distribution with mean V_e and variance σ_e^2 (in our device, $|e\rangle$ and $|f\rangle$ were difficult to distinguish). Then, for each measurement, instead of recording a binomial outcome $A = \pm 1$, we actually record a continuous value $\frac{2V-(V_g+V_e)}{Vg-V_e}$ that is normal-

ized to get a final unbiased estimation. The readout error increases the variance in the Wigner measurement and therefore the total sampling overhead. Based on experimental data, we choose $\frac{\sigma_g}{|V_g - V_e|} = 0.37$ and $\frac{\sigma_e}{|V_g - V_e|} = 0.35$ for our simulations. If we choose $V = \frac{V_g + V_e}{2}$ as the threshold to distinguish $|g\rangle$ or non-$|g\rangle$ outcomes, this can also be understood as the qubit in $|g\rangle$ having an 8% chance to be misidentified as non-$|g\rangle$, while the qubit in $|e\rangle$ or $|f\rangle$ having a 9% chance to be misidentified as $|g\rangle$.

Continuing to the OLI simulations, we follow the protocol introduced in Ref. [1] to reconstruct the state by measuring the real part of generalized M-mode Wigner functions $\mathrm{Re}[\tilde{W}_\rho(\vec{\boldsymbol{\alpha}}, \vec{\boldsymbol{\theta}})] = \mathrm{Tr}[\rho D(\vec{\boldsymbol{\alpha}}) \cos(\sum_{m=1}^{M} \theta_m a_m^\dagger a_m) D^\dagger(\vec{\boldsymbol{\alpha}})]$. We reconstruct the state within the 2^M dimensional subspace spanned by $\{|0\rangle, |1\rangle\}^{\otimes M}$. To find a set of displacement vectors $\vec{\boldsymbol{\alpha}}$, for each mode (with given θ_m), we generate a dataset $\{\alpha\}_m$ which produces minimal condition number [9] in single-mode Wigner tomography. We construct 384–393216 $\vec{\boldsymbol{\alpha}}$ vectors, where each component α_m of the vector is randomly chosen from its corresponding dataset $\{\alpha\}_m$. To simulate the Wigner measurement, we again sample a binomial variable 10 times with probability P_g (defined in Supplementary Eqn. (33)) to get $A = 1$ for $\phi = 0$, and another 10 times with P_g for $\phi = \pi$. We also model the readout error as described above. Finally, we use linear inversion to obtain the reconstructed density matrix and apply the algorithm in Ref. [8] to get a physical result. The OLI results for the fidelity and F-norm distance with respect to the ideal $|W\rangle\langle W|$ state are shown in Supplementary Fig. 3(b) and 4(b). The Total Measurement Number is defined as the number of sampled $\vec{\boldsymbol{\alpha}}$ vectors times 10 averages per $\vec{\boldsymbol{\alpha}}$ vector.

As shown in Supplementary Fig. 3, the resulting infidelity vs. total measurement number for each M is fit to a power law, and the intersection with 0.1 (90% fidelity) is used to generate the values plotted in Fig. 1(b) in the main text. We can see that the OLI method requires fewer measurements than DEMESST for 2 modes, but DEMESST has a lower sampling requirement for 3 or more modes. This effect becomes increasingly apparent for larger M. Based on the theoretical results from Supplementary Note 4 and discussed in this Note, DEMESST scales polynomially with M, while OLI scales exponentially with M. Similarly, in the F-norm distance plots in Supplementary Fig. 4, we again see that the DEMESST method performs increasingly more efficiently as the mode number increases.

Comparing to the experimental data presented in the main text, the matrix distance results are similar, albeit with some differences. For example, we can see that the measurement number at which the simulated distance reaches roughly 0.1 for 2 modes is slightly less than 10^5, while we observe a distance slightly above 0.1 at that point number in our data. For the 3-mode case, in the experiment we observe a distance of roughly 0.3 at 10^5 measurements for DEMESST and 0.4 for OLI, which is close to the simulated distances of roughly 0.25 and 0.3, respectively. For the 4-mode case, we measure a distance of 0.4 at roughly 2×10^5 measurements for DEMESST, compared to a simulated distance of roughly 0.2, and a distance of roughly 1.0 vs 0.8 for OLI at that measurement number. We attribute the discrepancies to imperfect state preparation and fluctuations over time in the readout error that may affect the accuracy of the simulation. This effect is particularly pronounced for the 4-mode case, where more measurements are required.

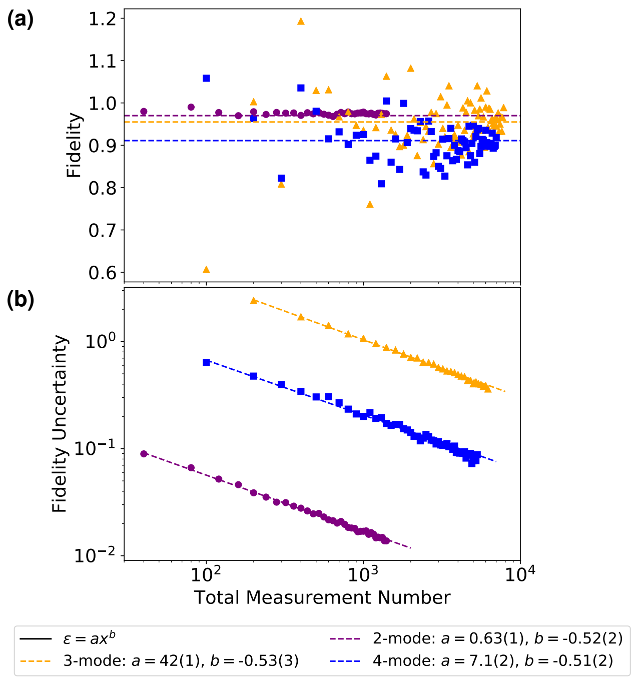

Supplementary Figure 5 | Experimental results for the W^2 tomography sampling method. (a) State reconstruction fidelities for 2- (purple circles), 3- (orange triangles), and 4-mode (blue squares) W states. Horizontal lines indicate the fidelities obtained from the OLI method, which are consistent with the W^2. (b) Error magnitudes for the fidelities shown in (a). All results follow a roughly $1/\sqrt{x}$ relationship vs. total measurement number, as expected.

Supplementary Note 8 – W^2 State Reconstruction

Another Wigner tomography sampling method that we implement is the W^2 method, which was first introduced in [5] and that we briefly discussed in Supplementary Note 3. In this approach, sets of coherent cavity displacements α_i are chosen using rejection sampling. This approach computes the overlap between a prepared state and a desired target state. For M modes, a cutoff c and a displacement vector $(\beta_1, ..., \beta_M)$ is randomly sampled from a uniform distribution between 0 and a maximum value of $|\mathcal{W}(\alpha_1, ..., \alpha_M)|^2 \prod_{i=1}^{M} |\alpha_i|$, where $\mathcal{W}$ corresponds

to the target state. If $|\mathcal{W}(\beta_1, ..., \beta_M)|^2 \prod_{i=1}^{M} |\beta_i| > c$, the vector is kept. This ensures that we measure cavity displacements that provide the most information about the state, while also avoiding displacements with large magnitude or Wigner values near zero, which are more susceptible to experimental errors. After measuring a set of n of these vectors, the final overlap fidelity is computed as $\frac{1}{n}\sum_{i=1}^{n} \mathcal{W}_{\mathrm{exp}}(\overrightarrow{\alpha_i})/\mathcal{W}_{\mathrm{ideal}}(\overrightarrow{\alpha_i})$. This approach allows for direct fidelity estimation of a prepared state compared with an ideal state. The W^2 method can also be used in a similar manner to DEMESST, where the fidelity estimation of a prepared state is performed with respect to an element of the multimode Fock state basis to determine the value of that matrix element. Repeating this for multiple different basis elements can thus provide a reconstructed density matrix.

Experimentally, we use the W^2 method as an additional check on our prepared W states. We set the target state to be the multimode W state with ϕ's determined from the DEMESST and OLI methods. The W^2 measurements then provide a direct fidelity estimation of the prepared state with the expected target. Since the sampling uses these angles, we present the W^2 results independently from the DEMESST and OLI, which do not utilize that information. The results are shown in Supplementary Fig. 5. The fidelities for the maximum provided observation number are 0.972 ± 0.013, 0.95 ± 0.35, and 0.90 ± 0.08 for the 2-, 3-, and 4-mode W states respectively. These averages are consistent with the results of the previous DEMESST and OLI methods, with the OLI fidelities indicated by the horizontal lines in Supplementary Fig. 5(a), and the data converges quickly to the expected fidelity obtained from those two approaches, although with large uncertainties, as shown in Supplementary Fig. 5(b). One reason for these errors is the relatively low total measurement number compared with the other methods. However, an odd behavior is that the 3-mode data has much greater uncertainties than even the 4-mode case, when we would expect the uncertainties to increase monotonically with mode number. Some possible explanations for this behavior could be a particularly low readout fidelity during data collection or fluctuations in drive strength during the measurement sequence that modify the effective Wigner operator differently for distinct sets of cavity mode displacements. This could also be caused by the choice in cutoff, as derived in Supplementary Note 3. All the uncertainties have the expected $1/\sqrt{x}$ with total observation number.

[1] Chakram, S. et al. Multimode photon blockade. Nature Physics **18**, 879–884 (2022).

[2] Chakram, S. et al. Seamless high-q microwave cavities for multimode circuit quantum electrodynamics. Physical Review Letters **127**, 107701 (2021).

[3] Cahill, K. E. & Glauber, R. J. Density Operators and Quasiprobability Distributions. Physical Review **177**, 1882–1902 (1969). URL `https://link.aps.org/doi/10.1103/PhysRev.177.1882`.

[4] Cahill, K. E. & Glauber, R. J. Ordered Expansions in Boson Amplitude Operators. Physical Review **177**, 1857–1881 (1969). URL `https://link.aps.org/doi/10.1103/PhysRev.177.1857`.

[5] da Silva, M., Landon-Cardinal, O. & Poulin, D. Practical characterization of quantum devices without tomography. Physical Review Letters **107**, 210404 (2011).

[6] Flammia, S. T. & Liu, Y.-K. Direct Fidelity Estimation from Few Pauli Measurements. Physical Review Letters **106**, 230501 (2011). URL `https://link.aps.org/doi/10.1103/PhysRevLett.106.230501`.

[7] Gertler, J. M., van Geldern, S., Shirol, S., Jiang, L. & Wang, C. Experimental Realization and Characterization of Stabilized Pair-Coherent States. PRX Quantum **4**, 020319 (2023). URL `https://link.aps.org/doi/10.1103/PRXQuantum.4.020319`.

[8] Smolin, J. A., Gambetta, J. M. & Smith, G. Efficient method for computing the maximum-likelihood quantum state from measurements with additive gaussian noise. Physical review letters **108**, 070502 (2012).

[9] Reinhold, P. Controlling Error-Correctable Bosonic Qubits. Ph.D. thesis, Yale University (2019). URL `https://rsl.yale.edu/sites/default/files/files/RSL_Theses/Reinhold-Thesis%20(1).pdf`.
